# Clinique Medicale, &c.

**Published:** 1835-04-01

**Authors:** 


### Clinique Medicale, ou Choix d'Observations recueillie a l'Hôpital de la Charité. Par G. *Andral*, &c. Deuxieme Edition, révue, corrigée, et augmentée par l'Auteur. Tome V. —Maladies de l'Encéphale. Londres: Dulau, Soho-square.

We have to apologize to our readers for having delayed, till now, our notice of the present volume of M. Andral's valuable work. In the former edition of the Clinique Médicale, it will be recollected that our author dedicated a volume to the subject of fevers. This he has not done in the present edition; all the cases, however, formerly contained under that head have been still retained, but differently arranged, some being referred to diseases of the abdomen, and some to the class of cerebral diseases. Thus the facts, though differently interpreted, still remain the same. From this change in the arrangement, we are inclined to suspect that M. Andral has joined the ranks of the Broussaiists—at least that he has renounced the doctrine of the essentialism of fevers. Whether he is right or wrong in having done so, we shall not now stop to enquire. We have already expressed our opinions on that subject freely, fully, and candidly. That any serious mischief can result with respect to the treatment of disease from thus localizing fever, particularly in this part of the world, where, whether from the nature of our climate, or habits of the people, or both, almost all febrile disturbance is followed by some local inflammation, we do not at all apprehend:—That Broussais' doctrine may have done some service in the treatment of fever, if in no other way than by inculcating the necessity of caution in the use of powerful emetics and drastic purgatives, and by frequently directing attention to the destructive effects of the calomel and jalap system, we are most willing to acknowledge—but what we do say, and what we think cannot be said too often, is this, that Broussais' doctrine, as well as the doctrine of all those who look for the causes of disease exclusively in organic lesion, no matter whether of the brain, lungs, or intestinal mucous membrane, must unavoidably narrow and restrict the views of the pathologist, and prevent the mind from ever extending its researches beyond the limited precincts of mere morbid anatomy. Thus the field of chemical pathology, which, if cultivated, holds out the most flattering hopes to the zealous investigator of disease, has been almost entirely neglected. We trust, however, that the æra is fast approaching, when due attention will be paid to the alterations produced by disease in the nature of the fluids of the body. To the exertions of such men as Berzelius and Prout, in this department of pathology, the profession is already much indebted. Were proofs necessary of the inadequacy of mere morbid anatomy to account for the phenomena of disease, we have it in abundance in the very volume we are now about to analyze. M. Andral says, p. 119—" Ce que vous voyez sur le cadavre ne peut donc pas toujours vous apprendre ce qui a eu lieu pendant la vie, et l'anatomie pathologique ne nous donne certainement le dernier mot, ni de la nature des maladies, ni de leur siége, ni de leur traitement."

The present volume, which now appears for the first time in a separate form, treats of diseases of the encephalon. It is divided into three books: the first contains diseases of the encephalic membranes—the second, diseases of the cerebrum—and the third, diseases of the cerebellum. Each book again consists of two divisions, the first containing cases to illustrate the phenomena and treatment, and remarks on the several particulars regarding the disease; whilst the second division consists of general deductions from the facts recorded in the first part, and exhibits a resumé of those results which the experience and observation of the author suggested. With the former of these divisions it is our intention to be very brief, and after noticing a few of the more striking cases, we shall pass directly to the consideration of those general results, the perusal of which is much more calculated to excite the interest and awaken the attention of the practical physician.

### Diseases of the Dura Mater.

Case 1.—*Fibrous Growth developed on the Internal Surface of the Dura Mater—considerable Depression of the Points of the Cerebrum corresponding to this Growth—Hemiplegia—the Intellect perfect—Headache of long standing.*

A military man, 61 years old, entered the Hôpital de la Charité in the commencement of the month of March, 1820. He appeared to have been originally of a good constitution; but at the time he presented himself for admission, he was very much reduced in flesh; countenance pale, approaching to a yellow. The right eyelid continued depressed by one-half before the globe of the eye, nor could it be completely raised by the patient at pleasure. The mouth quite straight, as was the tongue, which also was capable of being protruded without difficulty. Intellect perfect. The upper and lower extremities of the right side were deprived of the power of motion, and their sensibility also gone. The urine was voided involuntarily; pulse very slow (scarcely fifty); no appreciable disturbance in the organs of digestion and respiration.

This person stated that, after having for a long time suffered rheumatic pains in different parts of the body, he was seized, towards the commencement of the year 1817, with a headache, seated principally about the anterior part of the left parietal bone. This pain was at first intermittent; it then became continued for an entire year; and finally, from the middle of the year 1819, it disappeared altogether. For a long time, the patient complained of no other disturbance in his general health, except this headache, which, at times, became insupportable. At the period nearly when it ceased, the upper extremity of the right side appeared to him somewhat more insensible than the left; from time to time, the fingers of this side seemed deprived of all feeling; they were cold, and presented usually a violet tint; by degrees, he became unable to hold any thing firmly in the right hand. Subsequently, the upper extremity of this side became completely paralysed, at the same time that the left lower extremity gradually lost the faculty both of sensation and of motion.

The state of the patient underwent no change during the first ten or twelve days after his entering the hospital ; but then his tongue became dry, pulse frequent, and his ideas confused; diarrhœa came on ; a large eschar formed on the sacrum, and the patient sunk towards the end of March, in an adynamic state.

*Post mortem Examination.* The vault of the cranium being raised, no appreciable lesion was found on the external surface of the dura mater. But when this was cut into for the purpose of examining the brain, it was found that, not far from the anterior extremity of the left hemisphere, it had contracted unusual adhesions to the subjacent parts. These adhesions were formed by cellular bands, which united the two layers of the arachnoid to each other. These bands circumscribed a spherical body, of the size of a large nut, which sank deep into the cerebral substance, to which, however, it did not adhere, being separated from it by a cellulo-vascular structure, which seemed to be the tissue of the arachnoid and pia mater, compressed by it. This body was attached by a small pedicle to the internal surface of the dura mater ; the fibres of this membrane were lost on the pedicle of the tumor, and could not be distinguished from its own proper tissue ; this tissue, which possessed considerable hardness, and a white tendinous appearance, was formed of fibres closely packed together ; they seemed to be a continuation of the fibres of the dura mater. The cerebral substance in contact with this tumor presented all its usual appearances. The other parts presented nothing very remarkable, except that the mucous membrane of the stomach, of part of the ileum, and of the colon, presented a bright red colour.

*Remarks.* The cerebral lesion, whose existence one would be most inclined to suspect in this case, both from the symptoms and progress of the disease, was unquestionably softening. At no period, however, was there observed that contraction of the limbs, which so often accompanies softening of the cerebrum; still it is one which frequently does not occur, and at other times is a symptom so transitory, that the patients themselves scarcely recollect to have felt it. The rheumatic pains which preceded the headache might have induced one to consider the latter, also, as depending on rheumatism. There is not a doubt but that pains of the head, similar to what occurred in the case now in question, have been taken for a consequence of neuralgia or rheumatism. This headache was here the first symptom, and it coincided, probably, with the commencement of the disease of the dura mater, and must have continued all the time that the inflammatory process, necessary for the formation of the adhesions which were discovered after death, was going on in the arachnoid around the tumor ; and it probably ceased, when the transformation of these adhesions into cellular tissue was complete. The gradual manner in which the paralysis developed itself is conformable to the nature of the affection ; it was not preceded by any loss of consciousness. No lesion explained the incontinence of urine. The injected state of the stomach, and of a part of the intestine, coincided, in this case, with the adynamic symptoms under which the patient sank.

Case 2.—*Osteo-fibrous Tumor, of the Size of a small Pullet's Egg, developed on the internal Surface of the Tentorium Cerebelli, to which it*

*adheres closely. Hemiplegia, with convulsive Movements at Intervals, of the Side opposite to where the Tumor exists. Atrophy of the Lobe of the Cerebellum corresponding to the Tumor. Death by Cerebral Hæmorrhage.*

A shoemaker, 47 years old, addicted to the use of alcoholic liquors, fell on the occipital bone one day that he was intoxicated, about four years before entering the La Charité. Immediately at the time of the fall, he experienced no uneasiness: he subsequently began to complain of a dull pain towards the left side of the occipital bone; considerable giddiness of the head occurred from time to time, which was sometimes attended by a momentary loss of consciousness. At a later period, of a sudden, the upper extremity of the right side became the seat of a violent and painful shock (secousse), as if tetanic. Five or six of these shocks succeeded each other rapidly, and for the three or four days following, the right arm remained benumbed, and rather weaker than that of the opposite side. At first, there were intervals of several months between these attacks; they afterwards returned more frequently, and at last re-appeared every ten or twelve days, constantly confined, however, to the right arm, and at the same time paralysis of this limb, at first temporary, became permanent. Gradually, the lower extremity of the right side also lost the power of motion; the giddiness of the head became so annoying, that he determined on entering the hospital. At this time his state was as follows: face and eyes injected—considerable embarrassment in his articulation—memory quite good—tongue straight, and put out with ease—mouth straight. Both sides of the face possess the same sensibility and the same power of motion. Complains of a dull pain towards posterior part of the head, both on the right and left side; the two extremities of the left side cannot move at the will of the patient—they have however, a certain degree of stiffness; pulse slow, but remarkably hard; impulse of the heart strong; the digestive functions seem perfect; tongue broad, moist, and free from redness; considerable embonpoint, and the muscular system still well-developed. On his admission into the hospital, when we were just about to bleed him, he was seized with all the symptoms of apoplexy, and died the following day.

*Post-mortem Examination.* In the site of the left part of the tentorium cerebelli, there existed a large tumor, which, on the one side, pressed the posterior lobe of the cerebral hemisphere of this side, and, on the other side, compressed the cerebellum. The structure of the cerebral hemisphere was not at all changed. The left lobe of the cerebellum was singularly diminished in volume, at the same time that its substance became unusually hard. Neither the cerebrum nor cerebellum were continuous with the tumor. The structure, &c. of this tumor resembled very much that described in the preceding case. The right hemisphere of the brain was the seat of a great effusion of blood, which, occupying at once the corpus striatum and optic thalamus, forced its way into the lateral ventricles through the septum, which was torn. The parietes of the heart were hypertrophied.

*Remarks.* The seat of the tumor in this case, as well as in the preceding, was in the dura mater, but in different points of it; in the present instance, it compressed the cerebellum, one of the lobes of which underwent considerable atrophy. Still none of those peculiar functional disturbances, which, according to authors, are dependent on lesions of the cerebellum, were here observed. No symptom here manifested itself, but what would have been observed had the seat of the tumor been in one of the hemispheres of the cerebrum. The paralysis occurred in the limbs opposite the side of the cerebellum wasted away by the tumor.

### Observations on Diseases of the Arachnoid and Pia Mater.

There are few diseases, whose symptoms present as many varieties, as acute inflammation of the meninges. Are there any well marked signs, by the aid of which we may readily distinguish, during life, inflammation of the membranes covering the upper surface of the brain from inflammation of the membranes lining the lower surface of this organ? Are there any specific functional disturbances appertaining to inflammation of the membrane lining the ventricles? by what signs may we recognize inflammation of the membranes covering the medulla spinalis? Whatever be its seat, can acute meningitis be distinguished by its symptoms, either from the other acute affections of the encephalon, in which this organ is found materially altered, or from the very frequent cases in which irritation of the brain, or of its membranes, purely sympathetic of irritation existing in some other organ, leaves no trace of its existence after death? In fine, what are the anatomical characters in the dead body, by the aid of which we can assert that there has really been acute meningitis in the cases where, during life, those symptoms existed which seemed to appertain to it? Such are the questions still undecided in medical science, to the solution of which our author thinks that the observations given by him may serve to contribute.

Case 3.—*Effusion of Blood between the Arachnoid and Dura Mater.* A coachman, 73 years old, of a strong constitution, had fallen from his seat nine years previously, and received a deep cut in the left temporal region. For this he was trepanned at the La Charité, since which he enjoyed perfect health. On the 20th March, 1822, he felt, without any known cause, in the right lower extremity, and in the arm of the same side, a numbness, with some difficulty in moving these limbs, pains in the elbow and ankle. At the same time he complained of vertigo, tinnitus aurium, headache and drowsiness. These symptoms gradually increased, and at last he became unable to attend to his usual business. Three days before entering the hospital, he began to feel some difficulty in moving the left lower extremity. He entered the La Charité on the 6th of April, 1822, when his state was as follows: obstinate constipation—tongue natural—the two extremities of the right side were still capable of performing some motion, but very feebly. The left lower extremity was somewhat weaker than the right—pulse full and strong. On the 8th, delirium set in. On the 9th, headache less, but the paralysis more marked, which continued gradually to increase. On the 13th, face very much injected; great drowsiness; hemiplegia of the left side incomplete—that of the right side almost complete; tongue red and dry; alvine and urinary discharges involuntary; pulse strong and frequent. 14th. The respiration stertorous—total loss of consciousness—coma, and, in the evening, death.

*Post-mortem Examination, 12 Hours after Death.* The arachnoid, thickened and red, was detached from the dura mater by effused blood, partly fluid and partly coagulated, which had completely dissected the serous membrane from above downwards, from the part contiguous to the great falx of the dura mater, to the temporo-parietal suture. *The effusion was greater on the left.* The depression of the hemispheres was nearly an inch on the left—only half an inch on the right. The sinuses contained a considerable quantity of blood.

*Remarks.* We here have a rare case of pathological anatomy; it is not easy to conceive how a delicate membrane, such as the arachnoid, can be separated from the dura mater by effused blood, without being torn. The predominant symptoms at first were in reference to the power of motion; there was double paralysis, as there was double effusion.

Case 7.—*Meningitis, confined to the Anterior Extremity of each Cerebral Hemisphere. Rosy Tint and some Softening of the Subjacent Grey Substance. Follicular Enteritis, proceeding towards a Cure. Symptoms of Ataxic Fever.*

This was the case of a boy, 17 years of age, who entered the La Charité on the 18th of February, 1824, with symptoms of slight continued fever; headache; tongue white and moist, slightly red at the tip and edges; thirst considerable; abdomen soft, and free from pain; constipation. For five or six days no change; but, on the 24th, there was observed an extraordinary look of distraction in the patient's countenance. On the day after, stupor was noticed; pupils dilated; tongue natural; pulse small and frequent; skin hot.—Twenty leeches to the neck. The stupor continued—extreme dilatation of the pupils, and then a remarkable sensibility of the skin. On 1st of March, more leeches to the neck, and blisters to the legs. Died on the following night.

*Post-mortem Examination.* Fulness of the veins on the convexity of the cerebral hemispheres. The pia mater covering the anterior extremity of each hemisphere very much injected, with the other changes mentioned at the heading of this case.

*Remarks.* Here was a case, where the inflammation of the membranes existed only in a very small extent of the external surface of the brain; the pia mater was injected only towards the anterior and superior part of each hemisphere; and in this same part, the grey substance of the convolutions participated, in some measure, in the irritation of the membrane covering them. And this is all that was found to account for the serious nervous disturbances which appeared towards the close of the patient's life. No doubt, however, but he died in consequence of the lesion of the nervous centres. But the entire of the disease did not reside there; when he entered the hospital, there was not the smallest appearance of their being affected; he merely had slight continued fever, without any well-marked local symptom; at that time the intestine was affected; the post-mortem examination shewed, however, that the enteritis was proceeding to a cure.

Case 12.—(p. 30.)—*Abuse of Alcoholic Liquors—Pleuro-pneumonia at first—Febrile Delirium—Purulent Infiltration of the Subarachnoid Cellular Tissue of the Convexity of the Hemispheres.*

This was the case of the driver of a cabriolet, 48 years old, who was addicted much to alcoholic liquor. He entered the La Charité on the 25th Sept. when he complained of a pain below the left breast, behind and low down on this side; in this part, a well-marked crepitating râle was heard. Cough and expectoration frequent; considerable fever; was bled from the arm, and leeched. On the 26th there was some amendment; but, on the 27th, he suddenly became delirious. This delirium still continued on the 29th, with pulse small and moderately frequent—tongue natural. The history of the case inclined us now to consider this affection to be delirium tremens, and we accordingly decided on the inutility of revulsives. We then had recourse to large doses of opium; 96 drops of Rousseau's laudanum were given in two doses. After taking the second dose, he fell into a tranquil sleep till the following morning, when he awoke, at the time of our visit, with his intellects quite clear; he fell asleep again. On the 1st of October, his reason still quite clear—face red, and pulse frequent—skin hot —the crepitating râle still heard on the posterior and left side of the chest; this state of the lung might account for the existence of the fever. The opium was now withheld. On the 4th, the fever again returned, the pulse became once more frequent, and the delirium manifested a disposition to return: however, all the unfavourable symptoms disappeared after a little time, and the patient appeared convalescent, and nearly fit to leave the hospital, when, on the 9th, the intellect again became disturbed; and though opium, and blisters, and sinapisms were employed, no amendment took place. On the 20th, the tongue, for the first time, lost its natural appearance: it became red and dry. All the symptoms continued to grow worse, and he died on the 27th.

*Post Mortem.* A turbid, milky liquid infiltrated the subarachnoid cellular tissue of the convexity of the hemispheres. The lateral ventricles contained a small quantity of limpid serum. Cellular bands united the pleura costalis and pulmonalis on the left side. A great quantity of colourless, frothy serum oozed out from the tissue of the lungs; this tissue retained the pressure of the fingers, like an œdematized limb.

*Remarks.* When this patient entered the hospital, he presented all the symptoms of pleuro-pneumonia. The inflammation was quickly met by active antiphlogistic treatment. Other symptoms then appeared, and suddenly a delirium supervened. For this, whilst the fever was still high, nearly 100 drops of Rousseau's laudanum was administered, in two doses; this practice was justified by the success which we had more than once found to attend the employment of opiates during the course of any other disease, in individuals addicted to spirituous liquors. The sudden improvement in this patient also seemed owing to the opium; he obtained sleep—his delirium and fever left him, and he seemed to be going on in a very satisfactory manner. Again the delirium and fever returned without any assignable cause; the good effects obtained from the opium in the first instance induced us to employ it again, but in a very small dose; and, whether it was owing to this circumstance, or that the functional disturbance of the brain was beyond the power of opium, the cerebral symptoms became every day worse, and in seven days from their first appearance the patient died. Now it is probable, that it was during these seven days that the alteration took place in the meninges. The milky infiltration of the pia mater was evidently the result of an inflammatory process in this membrane. Was it already inflamed at the time that the opium succeeded in putting a stop to the delirium? Yet how could opium check or remove a sanguineous congestion of the cerebral membranes, when we know that, if given in a certain dose, it most commonly produces such a congestion, or at least symptoms which are explained by this congestion? However, it is not necessary that congestion should have existed in the membranes in order to produce the delirium, which was removed by the first dose of opium. Delirium has been frequently observed, in cases wherein, after death, there was no such lesion found as meningeal congestion, no lesion, at least, appreciable to the morbid anatomist. M. Andral here asks whether opium, which is so injurious when once the sanguineous congestion is established, may not be then employed; and whether, if administered to an individual whose brain is no longer in its normal state, it does not lose the property of exciting the congestion which it would give rise to, if the brain were in its healthy state. He thinks it probable, that if the opium, in this case, had been administered in as large a dose the second time as it had been at first, the moment the delirium began to re-appear, the nervous symptoms would have been removed the second time also.

Case 21.—(p. 59.) This is the case of a man, 50 years of age, who entered the La Charité with considerable anasarca and ascites. No sign of organic disease of the heart was detected; respiration not embarrassed; never complained of any pain in the right hypochondrium. He told us that the swelling had commenced about three months previously. After having been about a fortnight in hospital, he was observed one morning to be deprived of sense and motion, and in a strictly apoplectic state; face pale, eyes sightless, pupils very much dilated, pulse not frequent, respiration hurried and stertorous. He died some hours after the visit.

*Post Mortem.* Great serous infiltration of the extremities. A moderate quantity of limpid serum infiltrated the subarachnoid cellular tissue of the convexity of the brain. The two lateral ventricles were confounded with the third, with at least two glasses-full of limpid serum, transparent as crystal. Lungs sound, as also the heart. A considerable quantity of limpid serum in the abdomen.

*Remarks.* The destruction of the septum appears to have been the result of mechanical pressure, made on it by the serum contained in the ventricles. This case comes strictly under the class of serous apoplexy. The patient, when entering the hospital, had no other disease but dropsy, the cause of which was then obscure, but which was after death accounted for, by the morbid condition of the liver. This individual had then a disposition to serous effusions, when suddenly, no doubt, the serous membrane lining the parietes of the cerebral ventricles exhaled a large quantity of serum, which produced all the symptoms of genuine apoplexy. In the same way, in some dropsical patients, great dyspnœa has been observed to come on suddenly, and death follows the constantly-increasing difficulty of respiration. The cause of this was found to be an effusion of serum, which took place suddenly into the pleuræ. In this case, neither the anasarcous infiltration nor the ascites were diminished by the effusion into the ventricles. In another case described by M. Andral, the abdomen was thoroughly emptied, and the limbs unloaded of all serous infiltration, a very short time before symptoms of apoplexy manifested themselves, which symptoms were produced as in the present case, by a sudden effusion of serum into the ventricles.*

Case 28.—(p. 80.)—*Spinal Arachnitis—Arachnitis of the Base and Convexity of the Brain.*

This was the case of a woman, 28 years of age, the mother of four children ; her feelings had been very much affected by some gross insults that had been offered to her; her menses were suddenly suppressed, and she was seized with a violent shivering. The following day, great heat of skin—a feeling of constriction at the throat—globus hystericus—bilious vomiting. On the fourth day, the hysterical symptoms disappeared. She then entered the La Charité, when she presented the following state:—Face flushed—eyes very bright, and, as it were, very animated—neck swollen—head bent back —trunk flexed to one side, and cannot bend forward without great pain— continued pain all along the vertebral column, which is aggravated by the slightest motion—respiration difficult, and accompanied with panting—skin hot and dry—tongue natural—abdomen soft and free from pain—constipation. She was leeched and blistered repeatedly, which at first seemed to do her some good—the blood, on every occasion, was inflamed ; however, all her symptoms subsequently became worse—the pain of head and back became very acute—her features quite altered—her answers to questions slow and uncertain—pulse small and quick—respiration short. On the tenth day from the attack, a cold and clammy sweat broke out on the face—eyes insensible to all external impressions—no sensation of pain produced by pinching the skin—subsultus so great that the pulse could not be felt—violent trismus—death.

*Post Mortem.* The spinal canal being opened, and the dura mater divided, a layer of whitish, opaque matter was found extended all along, from the occipital foramen to the sacrum; on pressing this with the finger, a turbid liquid flowed into the cranium, mixed with little lumps of albumen ; on passing the scalpel over this layer, the instrument glides over it, and takes up nothing, which seems to indicate the existence of a membrane over this layer. On detaching the arachnoid from the internal surface of the dura mater, it was found that the diaphanous membrane covering the purulent layer is but a continuation of it, that portion of the arachnoid which, under ordinary circumstances, lines the pia mater, and which is here separated from it by a layer of purulent matter ; here, then, the pus is effused, not into the cavity of the serous membrane, but on its external surface, into the cellular

* The writer of this analysis met a case similar to the above; it occurred in Dublin about three years since. It was that of a man of very intemperate habits, whose liver was considerably enlarged ; he had ascites and anasarca, and was treated in the usual way, without any amendment. One morning, when he awoke, he found the size of the abdomen very much diminished, and the swelling of the lower extremities quite gone :—He arose from bed at 12 o'clock in the day, sat a few hours at the fire, became drowsy, threw himself on the bed, and when his wife went to call him, at about 4 o'clock in the evening, she found him dead. The ventricles of the brain were found to be enormously distended with limpid serum.

tissue uniting it to the pia mater. On the brain, the arachnoid and pia mater were very much injected towards the fissure of Sylvius. On the right side there was found an albuminous concretion, similar to that which filled the vertebral canal. Concretions similar to the preceding were found under the tentorium cerebelli. The lateral and third ventricles were distended with a milky serum in great quantity. Thoracic and abdominal organs sound.

*Remarks.* This case presents a combination of different symptoms, which are strikingly characteristic of acute inflammation of the spinal membranes. It was not, however, by these symptoms that the disease commenced; it perhaps, at first, was but a mere neurosis. M. Andral thinks that certain inflammations are preceded by mere nervous disturbance, in which, at first, all the disease consists, and that narcotics, administered at that moment, are very successful in dissipating all the symptoms; but allow the disease to proceed, and it will soon change its nature, and those functional disturbances, which at first depended on innervation, will be afterwards produced and kept up by inflammation, after which period narcotics would be mischievous. It was only on the fourth or fifth day that the nature and seat of the disease became manifest. The intelligence remained for a long time unaffected, whilst the power of sensation and motion was seriously interfered with. The pulse continued frequent; the dyspnœa, also, was constant and very sensible, which M. Andral thinks may be accounted for in this way, viz. that the primary seat of the disease lay in the spinal cord, in which he includes the medulla oblongata. A further reason for considering the affection purely nervous, is the moral cause which occasioned the menstrual suppression, and which was subsequently followed by the development of the severe symptoms.

### Lesions of the Dura Mater.

Lesions of the dura mater are much more rare than of the other membranes of the brain.

In the first and second cases above cited, we have remarkable instances of tumors developed on the internal surface of the dura mater, one being situated in that portion of the membrane in contact with the vault of the cranium—the other in the tentorium cerebelli. The texture of these tumors resembled that of the dura mater itself. In the first of these cases, no satisfactory cause could be assigned for the disease; in the second, it seemed to be the consequence of external violence done to the occipital region, whereby the tentorium cerebelli became the seat of that osteo-fibrous growth already described.

*Lesions of the Arachnoid.* Lesions of this membrane, as of other serous membranes, refer principally to its products of secretion. M. Andral cautions us against admitting the arachnoid to be the seat of a morbid secretion, unless the product of this secretion be found in its own cavity. These morbid products, however, are more frequently found outside the arachnoid, in the cellulo-vascular tissue constituting the pia mater. The morbid products found in the cavity of the arachnoid are the following:

1. An effusion of clear and transparent serum. Such an effusion is very rare on the upper surface of the brain; it is much more common at the base.

2. An effusion of turbid milky serum, with purulent flocculi. M. Andral saw but one instance of this in the cavity of the arachnoid.

3. False membranes, not yet organized, lining one or other of the free surfaces of the arachnoid.

4. False membranes of longer standing than the preceding, of serous organization, extended over one of the free surfaces of the arachnoid.

5. Adhesions of a cellular appearance, analogous to the bands of the pleura, and extending from the one to the other of the free surfaces of the serous membrane.

In some cases, the only morbid appearance is a remarkable dryness of the arachnoid, on the surface not adhering.

Either with or without these latter morbid products of secretion, M. Andral states that he never detected, in the arachnoid membrane, the slightest vascular injection, change of colour, or thickening. Where, at first sight, the arachnoid did appear either coloured or thickened, he thinks that the seat of this lesion is in the subjacent cellular tissue. Whatever be the nature of the membrane lining the ventricles, it presents, in the pathological state, nearly the same lesions as the arachnoid investing the brain; but these lesions are more frequently found in the former. It is, for instance, much more common to find serum effused, in considerable quantity, into the ventricles, than into the great cavity of the arachnoid covering the convexity of the hemispheres. The presence of this effusion in the ventricles should never, according to M. Andral, be considered as the result of a morbid process, except the quantity of it exceeds one ounce in each lateral ventricle. There is seldom observed any notable difference in the quantity of serum contained in each ventricle.

Instead of limpid serum, there is sometimes found in the ventricles a turbid liquid similar to whey, in which float those albuminous flocculi, as they are called, which constitute so frequent an anatomical character of pleuritis or peritonitis. In some cases the ventricles have been found filled with pus, which, by reason, no doubt, of its great weight, was accumulated principally in the lower part of each lateral ventricle. In most of the cases where pus has been found within the ventricles, some has also been detected in certain points of the subarachnoid cellular tissue surrounding the nervous centres.

*Lesions of the Pia Mater.* These have been more frequently observed than lesions of the other membranes. Our author gives the following classification of them:

1. Infiltration of its tissue by clear, colourless, transparent serum.

2. Infiltration of its tissue by a turbid, milky liquid, and sometimes by real pus.

3. A state of scirrhous induration of the tissue of the pia mater.

4. Serous cysts, variable in size and number.

5. Cartilaginous or bony structures, which M. Andral saw, in one instance, cover, like a second vault, the anterior fourth of the convexity of one of the hemispheres of the brain.

6. Tubercles, sometimes few in number, and scattered over a large surface, sometimes numerous and crowded together, forming by their union homogeneous whitish masses. It often happens that the tubercular matter is deposited between two convolutions, the space between which it perfectly fills up.

7. Adhesions, which are sometimes formed between the portions of the pia mater, which quit the arachnoid for the purpose of lining the interior of an anfractuosity, which then completely disappears, and several of the convolutions seem as if they were soldered together.

According to M. Andral, most of the lesions referred by medical writers to the arachnoid, and which are considered by them as the anatomical characters of arachnitis, are much more frequently seated in the pia mater. In almost all the cases, for instance, where the convexity of the cerebral hemispheres is covered with a layer of serum or pus, this layer has its seat beneath the arachnoid; by passing the back of the scalpel over the latter membrane, the morbid product is displaced, but not taken up. Every time that tubercular matter has been found by our author deposited around the nervous centres, or in their investing membranes, it was not the arachnoid that appeared to him to contain this morbid product, but the tissue of the pia mater. The same, he thinks, may be said of those cartilaginous or bony concretions occasionally found, in the form of grains, around the substance of the brain or spinal marrow. It is also in the pia mater that those small bodies are found, commonly known by the name of *glandulæ Pacchioni*, which, in some subjects, are very numerous towards the edge separating the upper from the inner surface of each cerebral hemisphere, the existence of which, however, is far from being constant. M. Andral considers those bodies, improperly called glands, to be a morbid product formed in the pia mater, and thinks that they have as little right to be deemed normal structures as those cellular bands in the pleuræ have, which some of the old anatomists looked on as physiological products in consequence of their frequent occurrence, and which they actually designated by the name of ligaments of the pleuræ.

Lesions of the pia mater may exist in different parts of this membrane. Their most frequent seat is on the convexity of the cerebral hemispheres. The pia mater investing the cerebellum, M. Andral has found to be less frequently changed in structure than that of the cerebrum.

With respect to the pia mater of the medulla spinalis, he finds, on consulting his own observations, as also those of other writers, that it is much less frequently the seat of morbid change than the pia mater surrounding the brain, properly so called, and also that, in most of the cases wherein the pia mater surrounding the cord has become the seat of purulent infiltration, this same infiltration is found in the encephalic pia mater. On the contrary, nothing is more common than to find the latter very much changed, whilst the other is perfectly healthy.

All possible changes of structure may exist in the membranes, without the substance of the brain itself at all participating in the change. Sometimes, however, the latter has been found changed at the same time as the membranes. Thus, in cases of inflammation affecting that portion of the membranes covering the convexity of the hemispheres, the grey substance of the convolutions has been found injected and softened. In some cases, in which the subarachnoid cellular tissue contained a great quantity of serum, M. Andral detected the existence of a species of œdema in the cerebral substance.

On slicing this substance, and pressing it between the fingers, a considerable quantity of a serous liquid was expressed from it, similar to that with which the pia mater was infiltrated.*

### DISTURBANCES OF THE FUNCTIONS.

These may be either of the functions of the organs of the life of relation, or of those of the life of nutrition. The former serve in a particular manner to characterize the disease; the latter, though not so characteristic, may also help in forming the diagnosis.

### DISTURBANCES OF THE FUNCTIONS OF THE LIFE OF RELATION.

*Lesions of Sensibility.* These may have as their seat, either the meninges themselves, or the different parts which receive their nerves from the cerebro-spinal axis.

Like all fibro-serous membranes, the membranes of the brain indicate most of the changes produced in them by a greater or less exaltation of their sensibility, whence results headache, one of the most important symptoms to be considered in the history of meningitis.

Our author now proceeds to consider the degree of frequency of this symptom in diseases of the membranes.

Out of twenty-eight cases of affections of the cerebral membranes detailed by him, sixteen complained of headache, and in the remaining twelve no such symptom was observed.

In the sixteen where the pain of head had existed, the alterations discovered on opening the bodies were the following:

In two of them (Cas. 1 and 2) tumors were found attached to the dura mater, which had compressed the nervous substance in contact with them.

In two others (Cas. 3, 4) there was an effusion of blood into the cavity of the arachnoid.

In two subjects (Cas. 20, 23) no other change was found than a considerable effusion of limpid serum into the ventricles of the brain.

Three other subjects (Cas. 6, 7, 9) presented merely a bright redness of the meninges. Another (Cas. 8) presented pseudo-membranous concretions on the interior of the great cavity of the arachnoid.

In five cases (Cas. 11, 17, 24, 26, 28) he found the pia mater, either of the convexity, or of the base, infiltrated with pus. In one of these (Cas. 26) there likewise existed cellular adhesions intimately connecting the two folds of the arachnoid covering the convexity of the cerebral hemispheres. This person had been all his life tormented with headaches.

In one case only (Cas. 18) the ventricles were found filled with a purulent fluid.

From these facts M. Andral concludes that the pain accompanying affec-

---

* This *cerebral œdema* was the only alteration discovered by M. Andral, on examining the body of an individual who, about 50 hours before his death, had fallen down suddenly, deprived of sense and motion. He died with all the symptoms characterizing a violent attack of apoplexy. That was an instance of what may be called serous apoplexy.

tions of the meninges may exist with diseases of these membranes, differing from each other both as to their nature and seat.

He next considers what were the lesions found in the meninges of the twelve, who complained not of headache (two of these cases he leaves out of the account for very just reasons).

In two of them (Cas. 10, 16) the pia mater was infiltrated with pus, either that portion of it covering the convexity (Cas. 10), or that covering the base (Cas. 16).

A sero-purulent liquid filled the ventricles in one subject (Cas. 19).

Simple serum, either effused into the tissue of the pia mater, or contained in cysts, was found on the convexity of the brain in three subjects (Cas. 13, 14, 15).

Without dwelling longer on this detail we see plainly that it follows from these facts that affections of the membranes, during which no pain of head was complained of, differed neither in their nature nor seat from those in which headache was a prominent symptom.

Out of sixty-two cases of acute inflammation of the meninges exempt from all other complication detailed in M. M. Parent du Chatelet and Martinet's Work on Arachnitis, there were fifty in which headache was a leading and prominent symptom.

Out of fourteen cases of meningitis described by Dance twelve complained of head-ache.

In the numerous cases of acute hydrocephalus published by Doctor Charpentier of Valenciennes, pain of head was a prevailing symptom.

Hence it follows that in the great majority of cases headache may be considered as one of the symptoms of acute or chronic affections of the meninges, and that it may accompany very different morbid lesions of these membranes.

M. Andral now asks whether this pain of head can by its presence serve to distinguish inflammation of the meninges from other diseases, which, though having their origin out of the brain, may however give rise to several of the symptoms characterizing acute meningitis? Such for instance as acute inflammations of the intestinal canal.

He states that of forty-five cases of inflammation of the digestive tube detailed in one of the preceding volumes of the present work, twenty one had decided headache—of the remaining twenty-four there were several the precise history of whose state was not accurately ascertained.

From his own observations, as well as from numerous cases of entero-mesenteric and typhoid fevers detailed in the writings of Dance, Petit and Serres, Bouillaud and Louis (in all which the only morbid lesion detected after death was in the intestinal canal), M. Andral infers that head-ache is a symptom met with in other cases besides those wherein the nervous centres are primitively and idiopathically affected; and consequently that it cannot be laid down, as a proof of the existence of an affection of the cerebral membranes. Being observed at the outset of many febrile affections, it indicates no doubt a disturbance of the innervation; but it no more proves the presence of real meningitis, than those painful affections of the limbs do, so common in such cases. M. A. conceives that it is to such pains, which are altogether nervous, and not at all connected with any inflammatory state of the organ in whose vicinity they are felt, we should attribute many of those epigastralgies so frequent at the commencement of fevers, and which he very properly thinks are too often and too lightly referred to a gastritis.

From several cases detailed in his own work, and very many described by other writers, M. Andral feels himself warranted in concluding that, though in many cases the seat of the lesion of the membranes is pointed out by that of the head-ache, it is however far from being always so.

With respect to the seat of the headache in continued fevers, he has found the pain in the very great majority of cases to be frontal, or suborbital; in some it is felt either at the temples, sinciput, or occiput; and in some cases the patient cannot point out its precise seat.

Thus, whilst there are some points of resemblance with respect to the seat of the head-ache in meningitis, and that in continued fevers, the pain is not in the latter so strictly confined to certain points of the head.

The intensity of the pain of head merits particular attention, when we wish to convert this symptom into a sign. The head-ache of continued fevers is scarcely ever made known to the physician unless he questions the patient particularly on the subject. On the contrary, in most cases of meningitis, the pain of head is the first and predominant symptom of which the patient complains.

Head-ache is almost the only modification of sensibility observed in the ordinary cases of meningitis. In some few cases, however, the sensibility of the skin has been found modified. This, however, M. Andral conceives to depend less on any specific morbid lesion, than on some peculiar disposition of the individual.

On comparing, with respect to their nature and frequency, the lesions of general sensibility, observed in cases of acute meningitis, with those seen in typhoid fevers, he concludes that these lesions are so similar in both, as to assist very little in the way of diagnosis.

Our author next considers how far and in what manner the organs of the senses are disturbed in meningitis, particularly the organs of sight and hearing. The modifications of the organ of sight were for a long time considered by pathologists as capable of serving to characterize certain acute or chronic affections of the brain.

These modifications may be classed under the three following heads:

1st. Modifications of the motions of the globe of the eye.

2d. Modifications in the state of the pupil.

3d. Modifications of vision itself.

The motions of the eye may be altered in several ways: sometimes they are irregular, and as it were convulsive: sometimes the globe is immoveable: sometimes there is strabismus, either of one side, or both. These different alterations cannot be referred to any specific lesion. They have been observed in adynamic and ataxic fevers without any appreciable lesion of the nervous centres. Strabismus, however, when permanent, our author considers to possess more value as a sign of meningitis, than either irregularity of motion, or immobility of the eyes.

The state of the pupil is far from being always the same in the different cases of meningitis. Several physicians consider that this opening, contracted and moveable in the first stage of the disease, becomes dilated and immoveable, as soon as serous or purulent effusion has taken place, either around the brain, or into the ventricles. This rule M. Andral considers to be any thing but correct; in as much as with lesions precisely similar, the pupils may present the most different appearance, and what is more, with lesions altogether dissimilar, these openings may present precisely the same appearance.

From a number of cases cited by our author, in some of which the pupils were contracted, in some dilated, in some a natural state of the pupil, and in others alternate contraction and dilatation, he concludes that it would be vain to attempt to connect such or such a state of the pupil with any specific morbid alteration of the membranes.

Vision itself is oftentimes disturbed in affections of the cerebral membranes. Sometimes it is lost altogether; sometimes it is perverted; in some cases diplopia has been observed; some persons cannot bear the rays of light on the retina. None of these alterations however can be said to belong peculiarly to meningitis.

The faculty of hearing has been, though rarely, affected in meningitis. M. Andral cites one case in which complete deafness was observed, in which the entire lesion discovered after death was seated on the upper surface of one of the lobes of the cerebellum, and consequently at a great distance from the origin of the auditory nerves. Deafness has been observed in cases of typhoid fever, where no morbid lesion whatever was discovered in any part of the brain to account for it.

An attentive consideration of the different alterations of sensibility presented by individuals labouring under meningitis, has convinced our author that none of these alterations are constant, none of them necessarily connected with such or such a form of meningitis, and that the disturbance of function is much less dependent on the membrane so affected, than on the brain itself. It is this organ which produces these disturbances, and consequently they must vary in the individual cases, according as the brain participates in, and sympathizes with, the irritation of its membranes. In this way also may be explained the infinite variability of the symptoms of pericarditis; for in this case too the determining cause of the symptoms is not in the pericardium, but in the heart itself. What we see on the dead body cannot always inform us what took place during life, and pathological anatomy will not certainly tell us all that is to be known with respect either to the nature, seat, or treatment of disease.

*Lesions of Motion.* These are met with more frequently in affections of the cerebral membranes, than lesions of sensation, but they are not more constant than the latter, and they are sometimes wanting in cases, where after death we find the same morbid changes of structure, as in the cases where they have existed.

Lesions of motion, observed in affections of the meninges, may be divided into two great classes; in the one, the motions continue, but they are performed in a disorderly way; in the other the motions no longer exist. The first class comprises the different spasms, which are sometimes clonic, sometimes tonic.

Under clonic spasms may be ranged those disturbances of motion, which have been observed in the different cases published regarding the diseases of the membranes.

Some persons present merely a sort of general agitation; they are constantly in motion, and rest appears to them insupportable; they are anxious incessantly to change their position.

In others this agitation is confined to some particular part of the body; some patients constantly move their arms or legs; in some the trunk is alternately raised and depressed; some constantly move the head from right to left, and from left to right. In these cases it is the will, though irregular, that produces the motion; there are cases, however, where the motions are involuntary; thus in some patients the muscles which terminate at the flexor tendons of the fingers, are agitated by violent contractions; hence subsultus tendinum; several present, as a prominent symptom, a tremor which is sometimes general, and sometimes partial.

Convulsions, properly so called, are among the most common phenomena accompanying acute meningitis. These are sometimes, though very rarely, general. When partial, they are sometimes confined to the same part, at other times they affect different parts of the body successively. The parts most usually affected with convulsive motions in meningitis are, the globes of the eyes, the eye-lids, the face, the lips, and finally the extremities.

Tonic spasms, as they are called, are not less frequent than clonic, in the disease now under consideration. Thus permanent flexion of the fore-arm on the arm is often observed. Reversion of the head back, its inclination to the right or left, are sometimes observed in cases of meningitis, as also tetanic rigidity of the neck, trunk, or extremities, trismus, &c.

Under the second class, in which motion is either diminished, or altogether destroyed, may be reckoned those numerous varieties of paralysis observed in meningitis. This paralysis may take place in the muscles of the eye, of the eye-lids, face, lips, or limbs: either one, or several of these, may be deprived of motion. In these different parts the paralysis may be established either slowly, or as instantaneously as the loss of motion succeeding cerebral hæmorrhage.

After considering in a purely nosological way the different lesions of motion in individuals attacked with meningitis, our author next proceeds to establish some relation between these functional lesions, and the different species of morbid changes found in the meninges after death. For this purpose he passes in review each of the disturbances of loco-motive action above-mentioned, first mentioning those cases only, where such disturbance existed singly at the last period of life.

With respect to *general agitation*, his own experience will not warrant him in saying any thing, as he never met that symptom existing singly in any case. In cases mentioned by others where this was a prominent symptom, the meninges covering the convexity of the brain were found injected and thickened. With respect to *partial agitation, subsultus tendinum*, and *trembling of the limbs*, his own observation will not enable him to say more, as he has not met these lesions existing singly. Having adverted to the other lesions of motion observed in affections of the cerebral membranes, he next considers the important phenomenon of *paralysis*. In the fifteen cases of hemiplegia observed by him, and to which he had previously alluded, he states that in four cases only (Case 1, 2, 4, 13) the brain was compressed by accidental productions developed in the meninges, which compression occurred, in every case, on the side opposite to that of the hemiplegia, and tended directly from above downwards. In five of these cases the compression existed on one of the cerebral hemispheres. In one only (Case 2) a tumor developed in the dura mater had compressed, and wasted one of the lobes of the cerebellum. In this last case also the paralysis was at the opposite side from the seat of the tumor, and nothing else was observed but simple hemiplegia, without any other modification of motion. However, before the hemiplegia was established, convulsive motions had existed in the arm of the side opposite to that of the lesion of the cerebellum: at a later period this limb lost altogether the power of moving, and ultimately the paralysis extended to the lower extremity of the same side.

In the subject of the fourth case the limbs of the right side became paralysed gradually; the fore-arm remained for a considerable time flexed on the arm. Blood was found effused on the left side between the arachnoid and dura mater.

Instead of hemiplegia some cases presented paralysis of a single limb. Thus in the fifteenth case detailed by our author, wherein the pia mater, traversed by tubercular granulations, was infiltrated with serum towards the convexity of the brain, the right arm, as also the right side of the face, were the only parts affected with paralysis.

He next proceeds to the consideration of those cases in which, instead of its being confined to some particular part, the loss of motion is general, and strikes both sides of the body either successively, or simultaneously. In all such cases, when the paralysis of both sides supervened before the last struggle, our author uniformly found in both sides of the brain lesions corresponding with this symptom.

The different facts which have been now analysed with the view of discovering what are the lesions, which, in cases of meningitis, coincide with the different alterations in the power of motion, lead M. Andral to this consequence; that, with similar lesions of structure in the dead body, the most varied disturbances in the function of motion are found to coincide; let there be convulsion or paralysis, in the greater number of cases the structural lesion after death will be the same. Thus beyond that lesion, which the scalpel points out as having its seat in the membranes of the brain, there is in the brain itself a modification not recognizable by the anatomist, which is produced no doubt by the lesion of the membrane, but which, variable in each individual, is the real cause of all the functional disturbances observed.

The different disturbances in the function of motion, now passed in review, may take place also in several other cases, where, the membranes being uninjured, the cerebral substance itself is altered. They also are observed in severe fevers, the seat of which we are so often induced by the symptoms to place in the brain. In fevers, as well as in acute meningitis, we often observe subsultus tendinum, convulsive motions, trismus, paralysis of different parts. But in the great majority of cases these phenomena are less frequent, less intense, and less durable. Their existence however proves that this same modification produced in the brain by the irritation of the membranes, may also be produced in it under the influence of other causes, and that, without there being found after death any appreciable alteration either in its substance, or in its investing membranes.

*Lesions of Intellect.* The intellectual faculties [illegible]ng found by M. Andral considerably interfered with in the great majority of the cases detailed in the present volume, he very naturally considers lesion of intellect a much more constant phenomenon in acute meningitis, than that either of sensation or motion. This alteration of intellect may present itself under two different forms in meningitis, either under the form of delirium, or of coma.

Delirium may be either violent and noisy, accompanied with a development of great muscular strength; or the patient may remain quite silent and still, exhibiting very great prostration of strength. Sometimes one single idea engages the patient; sometimes ideas of the most heterogeneous kind occupy his mind.

In some this disturbance of intellect attains its highest degree from the very commencement; in others it comes on gradually and insensibly. On reviewing the numerous varieties of delirium in the several cases described, M. Andral concludes that no single one of these various forms characterizes meningitis, that there is not one of them which may not be found in the different cerebral irritations which are purely sympathetic and unaccompanied with any structural alteration of the membranes appreciable on the dead body.

When once the delirium has shown itself it may not cease, presenting merely alternations of exacerbation and remission; it may also be only transitory. In some patients the delirium first lasts for a very short time; then at the end of a period more or less long it returns; the intermissions become more and more short, and at last the affection becomes continued. In some it appears only at night, and the clearness of the intellect during the day seems to exclude the idea of a meningitis altogether. In some a delirium of several days' duration suddenly disappears a little time before death, when the other symptoms become more aggravated.

Wherefore when endeavouring to distinguish the delirium of meningitis from delirium produced by sympathetic irritation of the brain, we would be wrong in laying it down that the latter only can be intermittent, as several cases prove, that the delirium arising from meningitis may be accompanied with perfectly lucid intervals.

The period of the disease when the delirium appears is far from being the same in every case. It hardly ever marks the very outset of the affection; so that when in the midst of perfect health delirium does appear, it is not at all probable, that it is the result of meningitis. In general, head-ache precedes it.

Out of forty cases of acute meningitis collected by M. Andral, in which he was perfectly certain both of the precise period when the disease commenced, and also of the moment when the disturbance of the intellect appeared, he found that the delirium developed itself.

| | | | |
|---|---|---|---|
| On the 1st day...... | in 3 cases. | On the 10th day...... | 0 |
| 2d.......... | 1 | 11th ........ | 0 |
| 3d.......... | 3 | 12th ........ | 1 |
| 4th ........ | 3 | 13th ........ | 4 |
| 5th ........ | 3 | 14th ........ | 1 |
| 6th ........ | 3 | 15th ........ | 2 |
| 7th ........ | 4 | 16th ........ | 1 |
| 8th ........ | 6 | 20th ........ | 2 |
| 9th ........ | 2 | 24th ........ | 1 |

Since with very few exceptions the delirium has manifested itself as a constant phenomenon in acute meningitis, our author fairly concludes that it may be produced in this disease, whatever may be the nature of the lesion of which the meninges are the seat. It is certainly curious to see how a simple sanguineous congestion of the pia mater, in those cases even wherein it is partial, or a little pus infiltrating its tissue, can produce the most serious disturbance of the intellect, whilst a more deep-seated alteration of the brain, an immense softening for instance, oftentimes will exist without producing the slightest disturbance of mind. Is it, as some have said, because the irritation of the membranes re-acts principally on the most superficial part of the brain, and that it is here particularly the intellect has its seat?

Instead of delirium, individuals labouring under meningitis may present a state of coma, which sometimes exists from the very commencement of the disease, and sometimes supervenes on delirium. The latter is much more frequent, at least in adults.

When a patient dies in the midst of a state of coma, the lesions found in the membranes differ neither in their nature, nor seat, from those found in cases where life is terminated in the midst of a state of delirium. Hence those authors are wrong, who say that delirium appertains exclusively to meningitis of the convexity of the brain, whilst coma is peculiar to that of the base. M. Andral thinks, that inflammation of the meninges, whatever be its seat, may at first determine in the brain a period of excitement, indicated by delirium, and then a period of collapse, either real or apparent, evinced by coma. These two stages exist in the great majority of cases which terminate in death. In some individuals, however, the stage of excitement is unusually prolonged, and they die before the coma has supervened; whilst in others, on the contrary, the phenomena of excitement are very transient, and the state of coma comes on without having been preceded by delirium properly so called. There are, in fine, some cases where coma is the first symptom; persons who a little before were in a perfect state of health, fall down suddenly deprived of sense and motion. Cases 21 and 22 of our author exemplify this apoplectic form of the disease. In these two cases nothing else was found, but an enormous distention of the ventricles by limpid serum; and in all such cases the ventricles have been uniformly the seat of the effusion. A case similar to the above is recorded by another physician; it was that of an old man, 76 years of age, labouring under organic disease of the heart. The dropsy symptomatic of this affection had gradually disappeared, and the patient appeared to be doing very well. However he got up one morning to go to the water-closet—returned quickly to his room with his face quite flushed, endeavoured to make towards his bed, when he fell down suddenly and expired without uttering a word.

In proportion to the greater or less rapidity with which the serous effusion takes place, very different forms of disease are observed. If a very large effusion take place from the arachnoid in a very short space of time, the result is a morbid state similar to what is produced by a very abundant cerebral hemorrhage, or in other words, apoplexy. This is serous apoplexy, an affection which the moderns have very improperly erased from nosological systems. If the serum accumulates somewhat less rapidly, either in the external pia mater, or in the ventricles, the symptoms are there observed to be such as accompany every irritation of the meninges, (see Case 20.)

If the accumulation of serum takes place slowly, there may result a third form of which an instance is presented in Case 22. Here the power of motion remains unimpaired, whilst the intelligence is weakened by degrees.

To conclude; in those cases where the disturbance of intelligence depends on an affection of the meninges, the cause of such disturbance can no more be referred to any specific alteration than that of motion or sensation can, and the diversity of the lesions of intelligence, as well as of those of motion and sensation, can only be accounted for by referring it to the different susceptibility of the brain to impressions.

### Disturbances in the Functions of the Organs of Nutritive Life.

Several of these disturbances are very important to be considered, when we would establish a diagnosis of meningitis, and distinguish it from other diseases, in which nearly the same functional disturbances of the brain are found. With respect to the digestive tube, for instance, there appears in several individuals, labouring under acute meningitis, very remarkable morbid phenomena, which supervene but very rarely in cases wherein the intestine is the seat of inflammation more or less intense. The circulation is equally disturbed in certain cases in so special a manner, that by combining the signs furnished by it then, with the signs given by disturbance of the cerebral functions, we may attain with certainty the diagnosis of a meningitis.

In a great number of persons attacked with meningitis, the digestive tube does not present, during life, any appreciable functional lesion, whilst, in others, it is the seat of disturbances more or less serious: in this latter case, it would be desirable to ascertain whether these disturbances are merely the result of an anormal influence, exercised by the nervous centres on the digestive canal, or whether they are dependent on an affection of this tube itself, an affection which has been superadded to the disease of the encephalic membranes.

Whenever the meningitis was not complicated with any other affection, M. Andral almost uniformly found the tongue to preserve its natural state; it is broad, moist, free from redness, and sometimes even paler than usual; most frequently it is covered with a light whitish coat. From a review of several cases, observed both by himself and others, our author makes this assertion, viz. that as often as in an individual, who died of meningitis, there was not found on dissection any morbid state, either of the digestive tube or urinary passages, the tongue never even once deviated from its natural state; it even preserved this state in several cases, where the lesions found in the stomach were of the class of those which most frequently produce no modification in the state of the tongue; and, in fine, it almost uniformly lost its physiological appearance only in cases wherein dissection pointed out lesions, which we know usually coincide with a more or less obvious modification of the state of the tongue.

As often, then, as in a patient, who presents several of the signs of encephalic irritation, the tongue is found red, dry, brown, &c. we shall be disposed to consider, either that this irritation is but sympathetic of another affection, or else that the latter is complicated with it. (There are, however, some exceptions to this rule; for in several of the cases detailed in the third volume of this work, wherein the predominant symptoms are those of *ataxic* fever, the natural appearance of the tongue might have induced one to infer the existence of meningitis; and yet there was no such affection, the entire lesion, as far at least as dissection could ascertain, being seated in the intestine.) Thus, among the numerous causes which may modify the state of the tongue, we must not rank meningeal inflammation.

In most of the cases of meningitis, where it was not complicated with any other affection, M. Andral has not found thirst to be a very prominent symptom. Loss of appetite often shews itself at the commencement of the disease; yet a desire of food is observed in some cases, after the intense headache which marks the invasion of the affection has already existed for several days.

In some, the epigastrium is the seat of pain, which is increased by pressure; yet M. A. has never, in any such cases, found a complication of gastritis. This epigastralgy shews itself near the commencement of the attack, and never becomes very intense.

Nausea and vomiting very frequently accompany acute meningeal inflammation; they almost always shew themselves at the commencement of the attack; sometimes they are so slight as to attract but little attention. (In Dr. Bright's Medical Reports, we see some cases in which vomiting was a very prominent symptom.) In those cases where nausea and vomiting have been observed, it frequently happens that we find no other symptom indicative of gastric disturbance; the epigastrium is free from pain, abdomen soft, and, when the case has terminated fatally, dissection can detect no gastric structural lesion, even though the vomiting had been very severe and obstinate. A striking instance of a modification of function, not accounted for by any change in the structure of the organ.

The part of the meninges inflamed has no influence whatever on the production of the nausea and vomiting.

Vomiting, when it presents the characters now described, when, for instance, a natural state of the tongue coincides with it, is a valuable sign to distinguish, at the outset, the nervous symptoms depending on idiopathic irritation of the encephalon from those which are connected with inflammation of the intestinal follicles.

*Lesions of the Circulation.* The disturbances produced by diseases of the membranes in the circulatory apparatus may regard, 1st, the motions of the heart—2dly, the manner in which the arterial expansions are performed—3dly, the capillary circulation—4thly, the qualities of the blood.

From a large number of cases of meningitis observed by M. Andral, in which he carefully noted the state of the pulse, with respect to frequency, he feels himself justified in concluding, that nothing is more variable than this symptom in individuals so affected. In some cases, he found the pulse accelerated—in some it was natural, and in others slower than ordinary. The latter variation, however, is of rarer occurrence than the others, in the cases observed by him, who, by the way, were all adults.

He observes, also, that the proportion of cases in which the pulse remains natural, or becomes slower than ordinary, is more considerable in acute meningeal inflammations than in those of the thoracic or abdominal viscera.

M. Andral makes here a remark of considerable practical importance, viz. that the slowness of the pulse becomes occasionally a sign of very great value, in distinguishing a real inflammation of the meninges from other morbid states which may resemble it; he here refers to some cases in which, the meninges becoming attacked during the course of some other affection, the commencement of this complication was marked by a striking change in the pulse, which having, up to that moment, been very much accelerated, suddenly lost its frequency. From the analysis of several cases, our author asserts that the absence of frequency in the pulse, no more than its slowness, depends neither on the seat nor nature of the meningeal inflammation; yet his general experience inclines him to think that this state of the circulation occurs more frequently when the ventricles are distended with a great quantity of liquid; the same phenomenon has been noticed where the ventricles were quite sound. With respect to acceleration of the pulse, this, he thinks, more particularly occurs in the cases, wherein the membranes covering the convexity of the brain are the seat of inflammation. With respect to the strength of the pulse in meningitis, it presents no constant character—in some cases it is hard, full, and vibrating, whilst in others it is small and concentrated from the commencement of the affection. It generally becomes weaker and more compressible during the period of coma. Irregularity in the rhythm of the pulse has been observed in some meningeal inflammations; but pathological anatomy is as unable to account for such a phenomenon as for its strength or weakness, frequency or slowness. The capillary circulation undergoes certain modifications in persons labouring under meningitis. These are recognizable principally in the conjunctiva and face; the commencement of the disease is frequently accompanied by great redness of both these parts; this state is sometimes succeeded by great paleness; sometimes this paleness exists from the commencement of the attack, and it continues to the very last moment of life, and this is observed not only in those cases where a copious effusion of serum is found after death, but also in cases where dissection detected proofs of very active inflammation in the membranes of the brain, such as infiltration of the pia mater with pus, flocculent serum in the ventricles, false membranes at the base of the brain. The temperature of the skin varies much in meningeal affections.

*Lesions of the Respiration.* This function does not always remain in its natural state, in individuals labouring under meningeal inflammation. The disturbances which it undergoes seem to depend on the influence exercised on it by the affection of the nervous centres, since, on dissection, no lesion is found in the lungs, sufficient to account for the change in the respiration. However, the cases in which the respiration remains unchanged in meningitis, are more numerous than those in which it undergoes modification. In those cases wherein the respiration has been modified, the anatomical lesions were completely insufficient to account for the variable influence which the irritated or compressed brain possesses over the respiratory apparatus. In a preceding volume of this work, cases are detailed in which the different modifications of respiration have been observed during life, without there being any appreciable lesion, either of the brain or its membranes:

any cause tending to disturb the innervation, either temporarily or permanently, is sufficient to produce this effect.

From the great length to which we find our notice of this interesting volume has extended, we find it necessary to reserve our analysis of the remaining portion for another number of the Journal. With respect to the merits of the book, it is almost unnecessary to say any thing in the way of eulogy or criticism. A work founded on inquiries so difficult, and conducted with such unwearied research, requires not the praise of the journalist to recommend it to the profession.

